# Supplementary material for: Murine SEC24D can substitute functionally for SEC24C during embryonic development
Source: Sci Rep. 2021 Oct 26;11:21100. doi: 10.1038/s41598-021-00579-x (PMC8548507; doi:10.1038/s41598-021-00579-x)
Supplement: Supplementary file 1 — Supplementary Information 1. [file 41598_2021_579_MOESM1_ESM.pdf]

Recombinase Source	G418 Sensitive	G418 Resistant	Mixed	Targeted Insertion	Random Insertions
				Sec24c$^{+/c\text{-}d}$	
**pDIRE** *(plates 1-3)*	31 (10.7%)	247 (85.8%)	10 (3.5%)	0	18/288 (6.25%)
**pCAGGS** *(plates 4-6)*	42 (14.6%)	157 (54.5%)	89 (30.9%)	1*	7/288 (2.4%)
**Total**	73 (12.7%)	404 (70.1%)	99 (17.2%)	1 (0.0017%)	25 (4.3%)

Table S 1. Summary of ESC co-electroporation results
Results of co-electroporation of *Sec24c*$^{+/GT}$ ESCs with pUC19-Sec24c-d and either pDIRE or pCAGGS-iCre and pCAGGS-Flpo. G418 resistance should indicate absence of recombination to remove the neomycin cassette present in the parental *Sec24c*^{GT} allele. The targeted clone identified (*=6-H9) contained a mixed population of G418 sensitive and resistant ESCs.

Primer Name	Sequence (5' to 3')
**Genotyping and Long Range PCR primers**	
A	AAGGCGCATAACGATACCA
C	TGAAGGCGCATAACGATACCACGA
D	CTGGCCTGACGCATAAGAGGGTGCCACACT
E	ACTAAGATGGGTCCACAAAAGAGC
F	GGTGGGAAGTCCACACTCTC
F9	CTTCGTATAGCATACATTATACG
G	GTACTAGGTGAGCCTGAAATCAATG
H	TCCTTCCCGTTTCCTCCTTAGCAT
I	TCGTACGTGGATTTCCTCTGCTGT
J	TGAGTTTGAGGTCCAACCTGGTCT
R	ATGGCTTCATCACGCCTAAGGGTA
R7	CCTATACTTTCTAGAGAATAGGAAC
S	GGTGCACAGATGTCTTACCCAGGAG
T	CATTCACACAGCTGCAGAATCCGCACTGATA
U	GAATCCGCACTGATAGCGCCGTCCGCCT
iCre10F	AGATGCTCCTGTCTGTGTGCAGAT
iCre10R	AGGGACACAGCATTGGAGTCAGAA
Flpo8F	TGATGAGCCAGTTCGACATCCTGT
Flpo8R	AGCATCTTCTTGCTGTGGCTGTTG
24c-GF4	CAGCTGATACTGATACTAGGATCCACGGAC
24c-GR4	GCACTGCTAACAGTTCGCTATTCCTTCCG
RAF5	CACACCTCCCCCTGAACCTGAAAC
**RT-PCR primers**	
qF1	GTGGGCAACCAGGACCTGCAGCCCCTGCTACTC
qR1	CTGGCCTGACGCATAAGAGGGTGCCACACTAT
qR3	TTTGCTGCAGCTGATAACCAGG

Table S2. List of primers used in this study All primer sequences listed 5' to 3'.

Primer Paris	*Sec24c*$^{+}$	*Sec24c*$^{c\text{-}d}$	*Sec24c*^{gt}	Type
C+D	--	563	--	Screening PCR
C+R	--	727	--	Screening PCR
F+H	--	689	--	Screening PCR
I+J	--	993	--	Screening PCR
S+T	--	484	--	Screening PCR
S+U	--	468	--	Screening PCR
F9+E	--	103	103	Screening PCR
A+R7	--	106	106	Screening PCR
G+E	308	--	308	Genotyping
F+E	--	568	--	Genotyping
qF1+qR1	--	266	--	RT-PCR
qF1+qR3	768	632*	--	RT-PCR
24c-GF4+U	--	7593	--	Long range PCR
RAF5+ 24c-GR4	--	5798	6647	Long range PCR

Table S3. Expected PCR products from various *Sec24c* alleles

Primer pairs listed correspond to assays carried out as either screening PCRs of ESC clones, genotyping of mice potentially carrying the *Sec24c*$^{c\text{-}d}$ allele, and long range PCRs confirming the genomic location of the modified *Sec24c* locus. *632bp product resulting from splicing around the *Sec24c-d* insertion into exon 5.
