# Supplementary material for: Murine SEC24D can substitute functionally for SEC24C during embryonic development
Source: Sci Rep. 2021 Oct 26;11:21100. doi: 10.1038/s41598-021-00579-x (PMC8548507; doi:10.1038/s41598-021-00579-x)

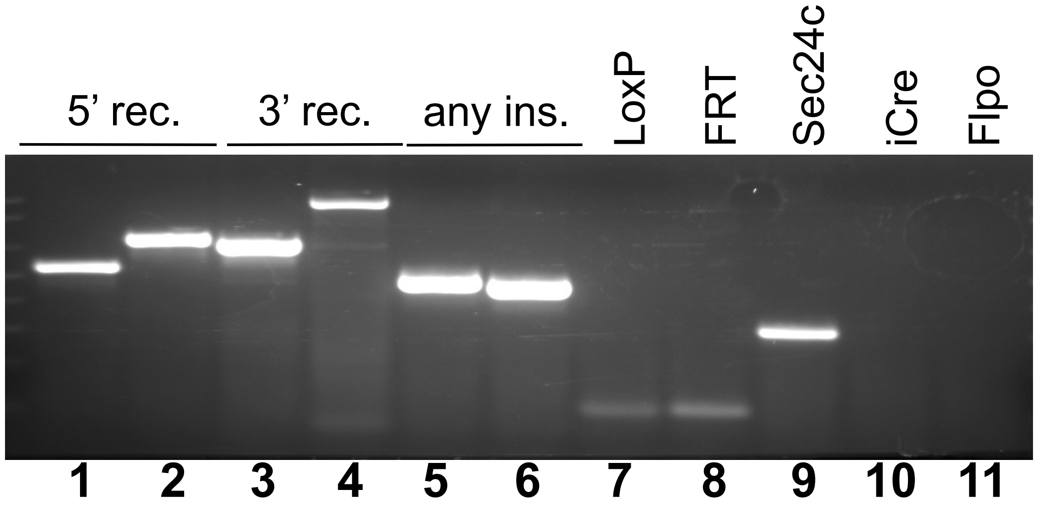

Figure 1C uncropped image (with 1Kb+ ladder)

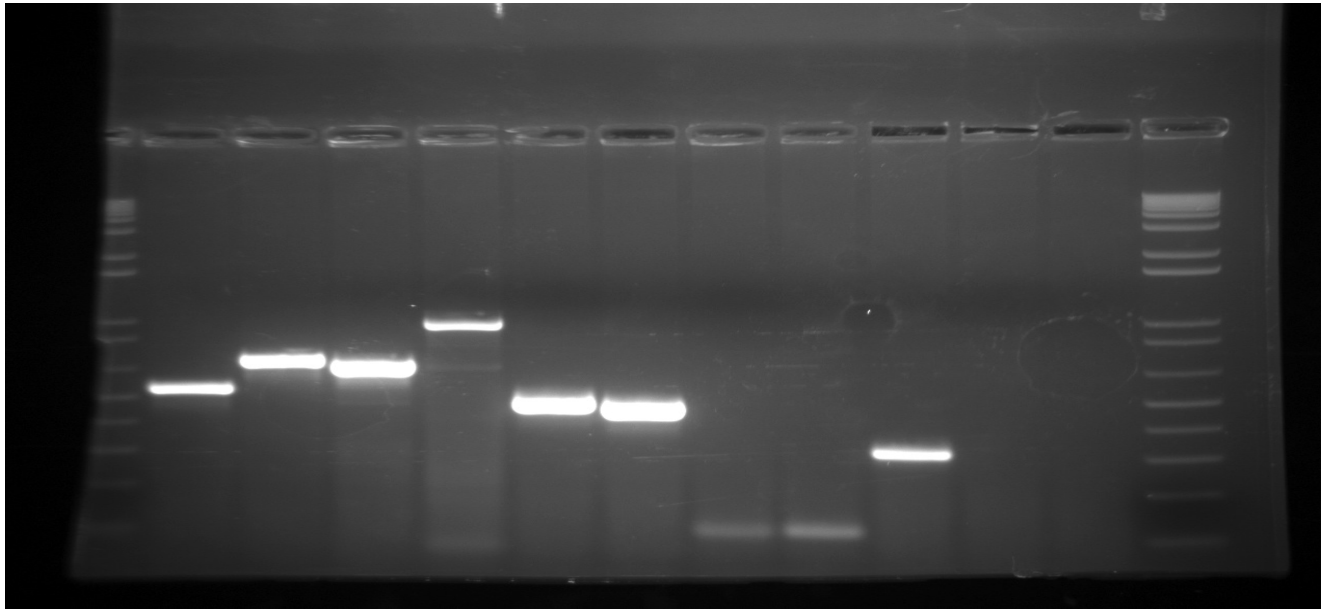

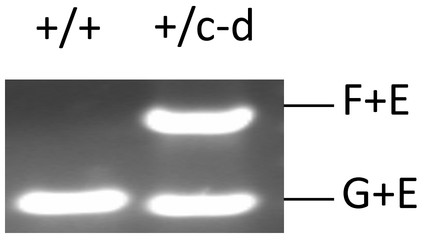

Figure 1D uncropped image (with 1Kb+ ladder)

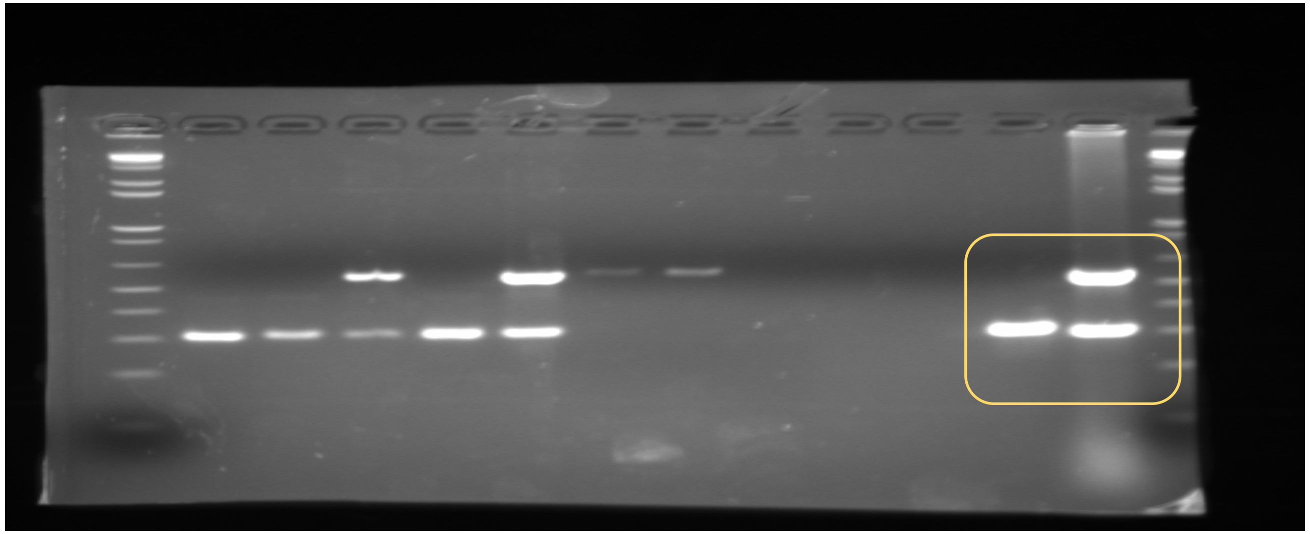

Figure 1E uncropped images (with 1Kb+ ladder)

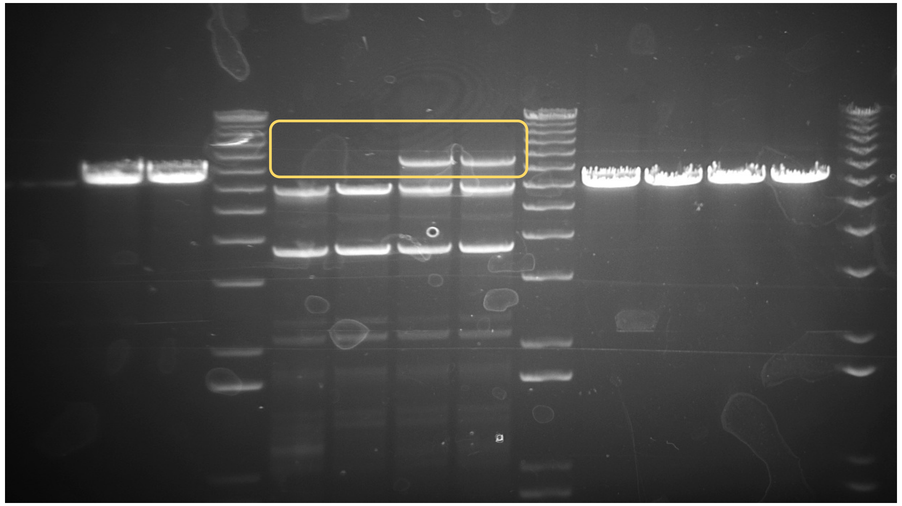

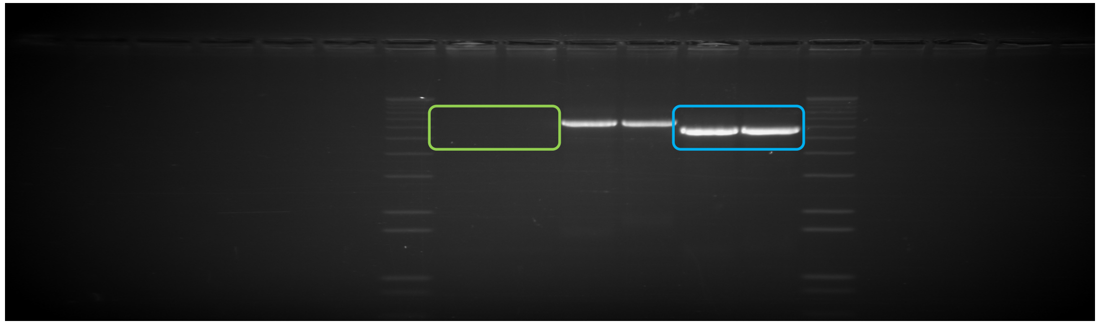

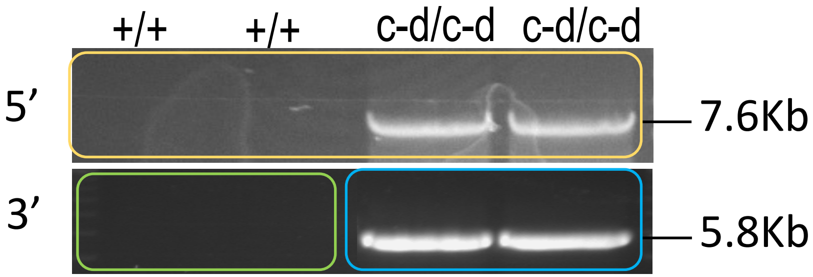

FIGURE 5

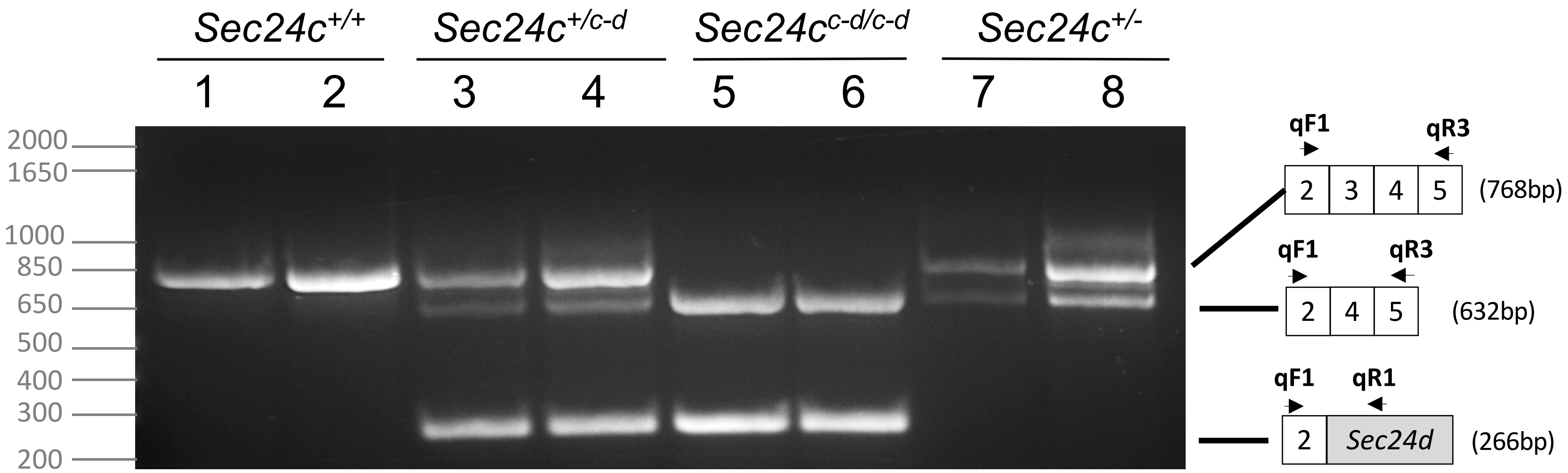

Figure 5 uncropped images (with 1Kb+ ladder)

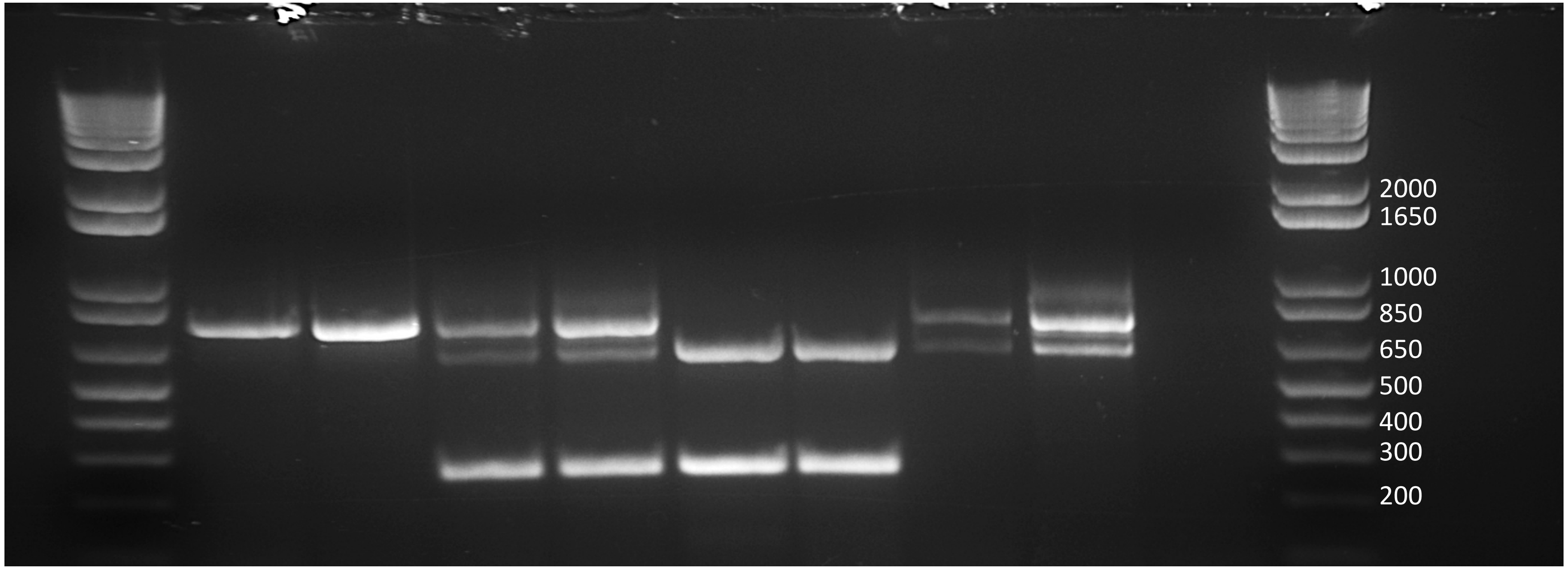

Supplement: Supplementary file 2 — Supplementary Information 2. [file 41598_2021_579_MOESM2_ESM.pdf]
